# Characterizing Diversity of Lactobacilli Associated with Severe Early Childhood Caries: A Study Protocol

**DOI:** 10.4236/aim.2015.51002

**Published:** 2015-01-01

**Authors:** Yihong Li, Silvia Argimón, Catherine N. Schön, Prakaimuk Saraithong, Page W. Caufield

**Affiliations:** 1 Department of Basic Science and Craniofacial Biology, College of Dentistry, New York University, New York, USA; 2 Department of Cariology and Comprehensive Care, College of Dentistry, New York University, New York, USA

**Keywords:** Lactobacilli, Early Childhood Caries, Bacterial Diversity, 16S rRNA, AP-PCR, Saliva, Dental Plaque

## Abstract

Lactobacilli have been consistently associated with dental caries for decades; however, knowledge of this group of bacteria in the etiology of the disease is limited to quantitative elucidation. Nowadays, explicit identification of oral *Lactobacillus* species is possible, despite their taxonomic complexity. Here we describe a combined approach involving both cultivation and genetic methods to ascertain and characterize the diversity and abundance of the *Lactobacillus* population in the oral cavities of children with severe early childhood caries (S-ECC). Eighty 3- to 6-year-old children (40 S-ECC and 40 caries free) who were seeking dental care at the Pediatric Dental Clinic of Bellevue Hospital in New York City were invited to participate in this study. Clinical data on socio-demographic information and oral health behavior were obtained from the primary caregiver. The data included a detailed dental examination, children’s medical history, and a questionnaire survey. Combined non-stimulated saliva and supra-gingival plaque samples were collected from each child and cultivated on selective media for quantitative measures of lactobacilli levels. The procedure for *Lactobacillus* species screening will include the random selection of 50 colonies per plate, extraction of DNA from each colony, and genotyping by arbitrarily primed polymerase chain reaction (AP-PCR). Each unique *Lactobacillus* AP-PCR genotype will be selected for taxonomic assessment by 16S rRNA gene sequencing analysis. *Lactobacillus* species will be identified by comparing the 16S rRNA sequences with the Ribosomal Database and the Human Oral Microbiome Database. Meanwhile, the same set of clinical samples will be independently subjected to genomic DNA isolation, 16S rRNA amplification with *Lactobacillus* genus-specific primers, sequencing, and taxonomic identification, both at genus and species levels with a customized pipeline. The distribution and phylogenetic differences of these *Lactobacillus* species will be compared between children with or without S-ECC. One of the main objectives of this study is to establish a study protocol for the identification and characterization of lactobacilli in the oral cavity. Future caries risk assessments can include lactobacilli counts (quantitative) and the presence/absence of specific cariogenic genetic signatures of a *Lactobacillus* species (qualitative) associated with S-ECC.

## 1. Introduction

Severe early childhood caries (S-ECC) is a particularly aggressive form of dental caries associated with a history of maternal malnutrition and illness, tooth developmental defect [1] [2], excessive exposure to carbohydrates [3] and early infection by cariogenic microorganisms, predominantly mutans streptococci (MS) and lactobacilli [4]-[6]. Evidence based on decades of studies indicates that MS and lactobacilli are involved in the initiation of dental caries [7]. Lactobacilli are associated with disease progression, as well as serving as an indirect indicator of the content of fermentable carbohydrate [4] [8]-[10]. Recent studies have demonstrated that the microbiota of children with S-ECC differs significantly from that of their caries-free counterparts [11]-[13] and that lacto-bacilli comprise a significant portion of the cariogenic biota [5] [14] [15]. High prevalence of lactobacilli in caries lesions and their ability to generate a low pH environment, as well as to survive in it, suggest that lactobacil-li are key determinants underlying the development and severity of caries, particularly in caries progression [5] [6] [11] [16] [17].

Studies have also demonstrated that the presence of lactobacilli alone in caries lesions or saliva is insufficient to make inferences about their specific contribution in caries development. Although more than 150 validly described species of *Lactobacillus* have been isolated from various human body sites, plants, foods and the environment [18] [19], only about a dozen species have been found in the human oral cavity, and the numbers are even fewer in caries lesions. It has been suggested that those *Lactobacillus* species are strong acid producers and may play a more important role in caries development than other lactobacilli [20]. Other studies suggested that a number of *Lactobacillus* species can be niche-specific colonizers that vary according to the environment [21] [22] and that certain *Lactobacillus* species found in caries lesions show more cariogenic characteristics than others [20] [23]. Thus, it is plausible that a subgroup of caries-associated lactobacilli possesses niche-specific genetic elements linked to caries development, to S-ECC in particular. However, the genetic and adaptive characteristics of these *Lactobacillus* species are unclear, as well as the mechanisms used by the *Lactobacillus* species to assert their dominance within carious lesions.

The lack of knowledge about the genetic characteristics and diversity of lactobacilli is, in part, hindered by the taxonomic challenges from the initial step of clinical sample collection to the final step of species identification. Traditionally, studies of caries-associated lactobacilli are based on laboratory cultivation, but results vary due to the lack of reliable protocols for screening and characterizing clinical isolates that enable the identification of lactobacilli. Recently, advancements in molecular genetics and comparative genomics have been applied to microbiology, largely facilitated by 16S rRNA sequencing and sophisticated analytical methods [24] [25]. It is now possible to quantify *Lactobacillus* colonization, identify lactobacilli to the species level, and determine the constellation of genetic elements associated with the cariogenicity of this group of bacterial species.

Previously, we reported a pilot study to demonstrate methods for determining the diversity of lactobacilli in the oral cavity [26]. The protocol proposed in this study updates some of those methods, streamlines the work-flow to minimize the time- and labor-intensive procedures from sampling to taxonomic identification, and incorporates a comparative genomics component. The study aims to: 1) establish a feasible protocol for sample collection, lactobacilli isolation, assessment of lactobacilli colonization and genotyping; 2) measure the diversity and abundance of lactobacilli in saliva and dental plaque using a culture-dependent approach; 3) measure the diversity of lactobacilli in saliva and plaque using a culture-independent approach, and compare it to culture-based diversity; 4) define the niche-specific genetic elements, *i.e*., genomic signature of *Lactobacillus* species/strains associated with S-ECC. The outcome variables can be included in future caries risk assessments, which, at present, rely mainly on lactobacilli counts in saliva. Monitoring the caries-associated genetic signature of some of the *Lactobacillus* species can also be clinically relevant for developing new caries treatment modalities.

## 2. Study Design and Methods

### 2.1. Design Overview

**Figure 1:**
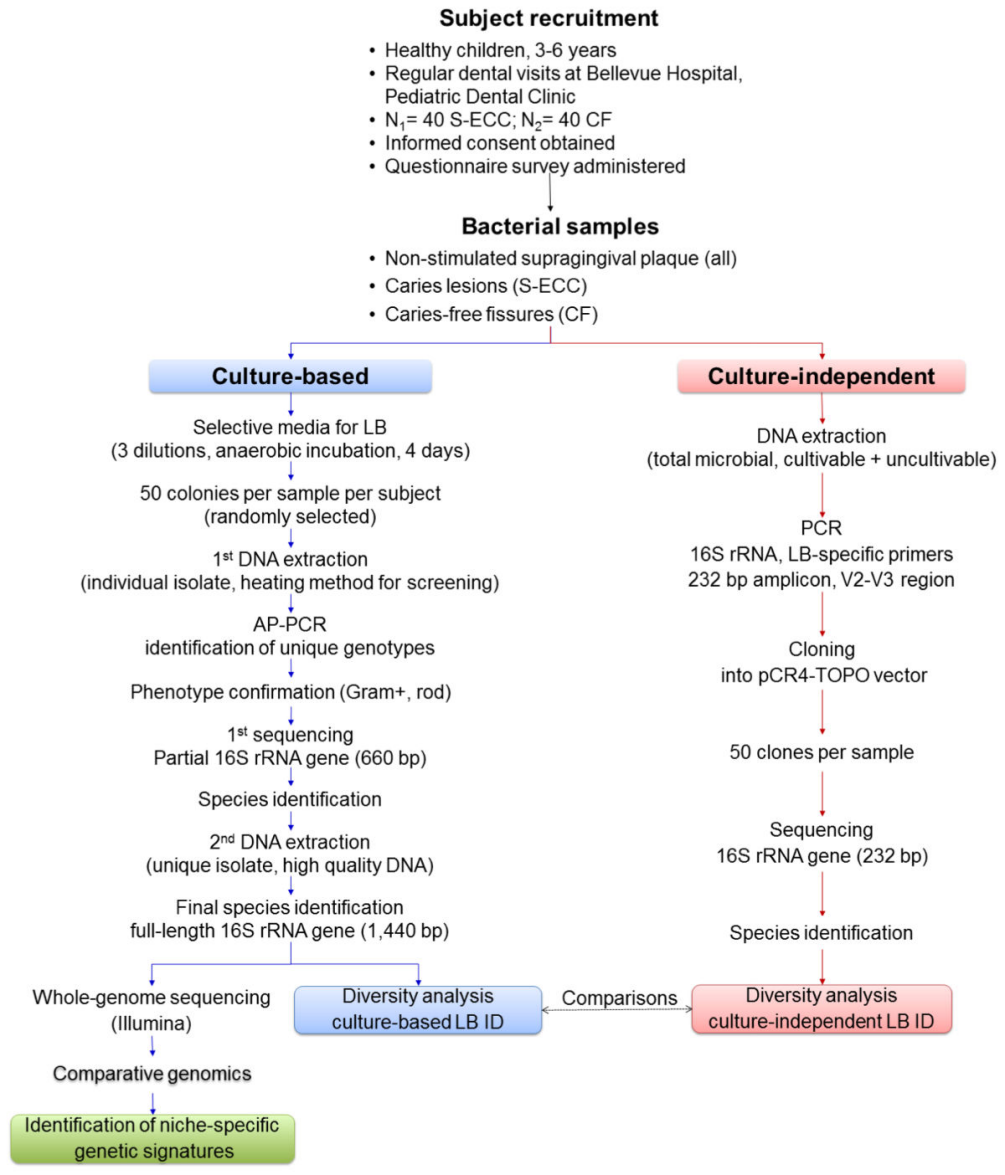
Schematic illustration of the overall study design. S-ECC = severe early childhood caries; CF = caries free; LB = lactobacilli; AP-PCR = arbitrarily primed polymerase chain reaction.

This exploratory study consists of four components: 1) subject recruitment, clinical and bacterial sample collection; 2) assessment of *Lactobacillus* abundance relative to other microbes; 3) determination of lactobacilli diversity at the species level in an aggressive S-ECC group and a caries-free (CF) group; 4) comparison of whole ge-nome sequences from the clinical *Lactobacillus* species/strains. The overall study design is illustrated in Figure 1. Our hypotheses are that: 1) lactobacilli colonization in S-ECC children differs in species abundance, distribution, and composition diversity when compared to that of CF children; 2) a subset of genetic loci in lactobacilli genomes constitutes a unique genetic signature that defines the caries-specific niche. The main objective of the study is to determine the characteristics of lactobacilli that play a key role in the progression and severity of early childhood caries. In order to accomplish the goal, both culture-based and culture-independent methods will be used to evaluate the level of lactobacilli in saliva and dental plaque, carious lesions and sound fissures and, consequently, to obtain individual *Lactobacillus* isolates. The final *Lactobacillus* species identification will be based on 16S rRNA gene sequence analysis. Furthermore, whole genome intra-species and inter-species comparisons will be conducted to identify niche-specific genetic elements (Figure 1).

### 2.2. Ethical and Governance Approval

The study is conducted at two sites: New York University College of Dentistry and the Pediatric Dental Clinic of Bellevue Hospital Center. Applications for this study have been approved by the Institutional Review Boards of the New York University School of Medicine, New York University College of Dentistry (Research Proposal Oversight Committee), and the New York City Health and Hospital Corporation (for Bellevue Hospital Center) for human subjects participating in research activities.

### 2.3. Subject Recruitment

The study recruitment began in February 2012 and ended in May 2014. A total of 80 children, 3 to 6 years of age, were recruited for this study from the Bellevue Hospital Pediatric Dental Clinic. Forty of them were diagnosed with S-ECC according to the criteria set forth by the American Academy for Pediatric Dentistry [27]. They were admitted to Bellevue Hospital Pediatric Dental Clinic for comprehensive dental treatment under general anesthesia in the operating room. A group of 40 caries-free children scheduled for regular dental checkup at the same clinic was recruited from the same Pediatric Dental Clinic at Bellevue Hospital as the comparison group. The parents or legal guardians of all children have given informed consent.

S-ECC is defined as caries in children younger than 3 years of age with one or more non-cavitated or cavitated lesions, filled or missing (due to caries) smooth surfaces in primary maxillary anterior teeth (dmfs), or a dmfs score ≥ 4 at age 4, ≥ 5 at age 5, or ≥ 6 at age 6 [27]. All of the S-ECC children were scheduled to be treated under general anesthesia at Bellevue Hospital. Caries-free (CF) children are defined as those children whose teeth showed no evidence of decay, treated or untreated, on any surfaces [28].

To be eligible for this study, children must be 3 to 6 years old, healthy without chronic diseases other than dental caries, and not having taken antibiotics within 3 months prior to bacterial sample collection. Children with chronic disease other than dental caries or a history of antibiotic use within 3 months prior to the dental treatment or missing more than 6 primary teeth are excluded.

### 2.4. Sample Size and Power Calculation

The study is designed to recruit 80 children divided into an S-ECC group (n = 40) and a CF group (n = 40). The sample size of estimation is based on data previously reported that the composition of oral microbiota of S-ECC children exhibits less diversity than that of CF children [12]. In an exploratory study of 15 S-ECC children and 8 CF children, we found that the mean similarity of the *Lactobacillus* population was 43.4% (SD ± 17.7%) for S-ECC children and 58.4% (SD ± 14.3%) for CF children, respectively (unpublished data). The proposed study hypothesis is that the *Lactobacillus* population between S-ECC and CF children is the same if the criterion for significance (alpha) is set at 0.05 for 2-tailed tests and the proposed sample size is 40 for each group. To test the null hypothesis, the study will achieve a power of >85% to yield statistically significant results.

### 2.5. Questionnaire Survey

Before undergoing dental examination, all primary caregivers who agreed to participate in this study were interviewed by a trained project coordinator to obtain the child’s current and past medical history, nursing practices, dietary habits, oral hygiene habits, and prescribed medications. Both English and Spanish versions of the questionnaire survey were available. The entire interview took no more than 20 minutes. Minimum or no risks result from participation in this study, and all information related to the project will be kept confidential following state laws and regulations governing confidentiality of medical records.

### 2.6. Oral Examination

A standardized clinical examiner performed the dental examination for each child in the Bellevue Pediatric Dental Clinic using standard dental mirrors and established criteria and scoring system. Using a sterilized soft toothbrush (Soft P-20 Oral-B Cat. 33259737, Henry Schein, Melville, NY), saliva and plaque samples were collected from all S-ECC children in the operating room by the standardized attending dentist. The same samples were collected from the CF children by a trained dental hygienist. A second bacterial sample was also obtained from caries lesions of the S-ECC children by the same dentist. More specific procedures of bacterial sample collection are described in the following sections.

### 2.7. Bacterial Sample Collection

#### 2.7.1. Supragingival Dental Plaque Mixed with Unstimulated Whole Saliva

1. Subject-specific
  - For S-ECC children scheduled for treatment under general anesthesia in the operating room: before comprehensive restorative treatment, the dentist brushed the child’s teeth for 1 min using a sterile soft toothbrush (Soft P-20 Oral-B).
  - For CF children in the dental clinic: the hygienist brushed a child’s teeth for 1 min using a sterile soft toothbrush without swallowing saliva.
2. Procedure
  - The dentist/hygienist slowly brushed the surfaces of the teeth, and then mixed with saliva.
  - The toothbrush was immediately placed into a pre-labeled glass tube containing 9 mL of a pre-reduced liquid dental transport (LDT) medium (custom order; Anaerobe Systems, Morgan Hill, CA).
  - The bacterial sample was removed from the toothbrush by shaking the toothbrush vigorously for 30 sec (approximately 30 strokes) in the LDT medium; the toothbrush was then discarded.
  - The bacterial sample was transported on ice to the microbiology laboratory within 2 h for processing and cultivation.

#### 2.7.2. Site-Specific Sample Collection

1. Caries lesion site-specific sample for S-ECC group
  - Bacterial sample was collected from 1 caries lesion at the most severe site.
  - A sterile regular No. 6 dental round bur (Dentsply International, Des Plaines, IL) was used with a slow-speed handpiece to remove caries lesion for 5 sec.
  - Bacterial sample along with the bur was immediately placed into a pre-labeled glass tube containing 1 mL of LDT medium (AS-916, Anaerobe Systems, Morgan Hill, CA), and the tube was capped.
  - The sample was transported on ice to the microbiology laboratory within 2 h for processing and cultivation.
2. Fissure site-specific sample for CF group
  - Bacterial sample was collected from 4 sound occlusal fissures (molar or premolar; 1 per quadrant).
  - A sterile 30-gauge extra-short dental needle (Henry Schein, Melville, NY) was held in a hemostat and gently passed across the 4 occlusal fissures to collect as much bacterial sample as possible.
  - The needle was immediately placed into a pre-labeled glass tube containing 1 mL of the LDT medium, and the tube was capped.
  - The sample was transported on ice to the microbiology laboratory within 2 h for processing and cultivation.

### 2.8. Culture-Based Assessment of *Lactobacilli* Colonization

**Figure 2:**
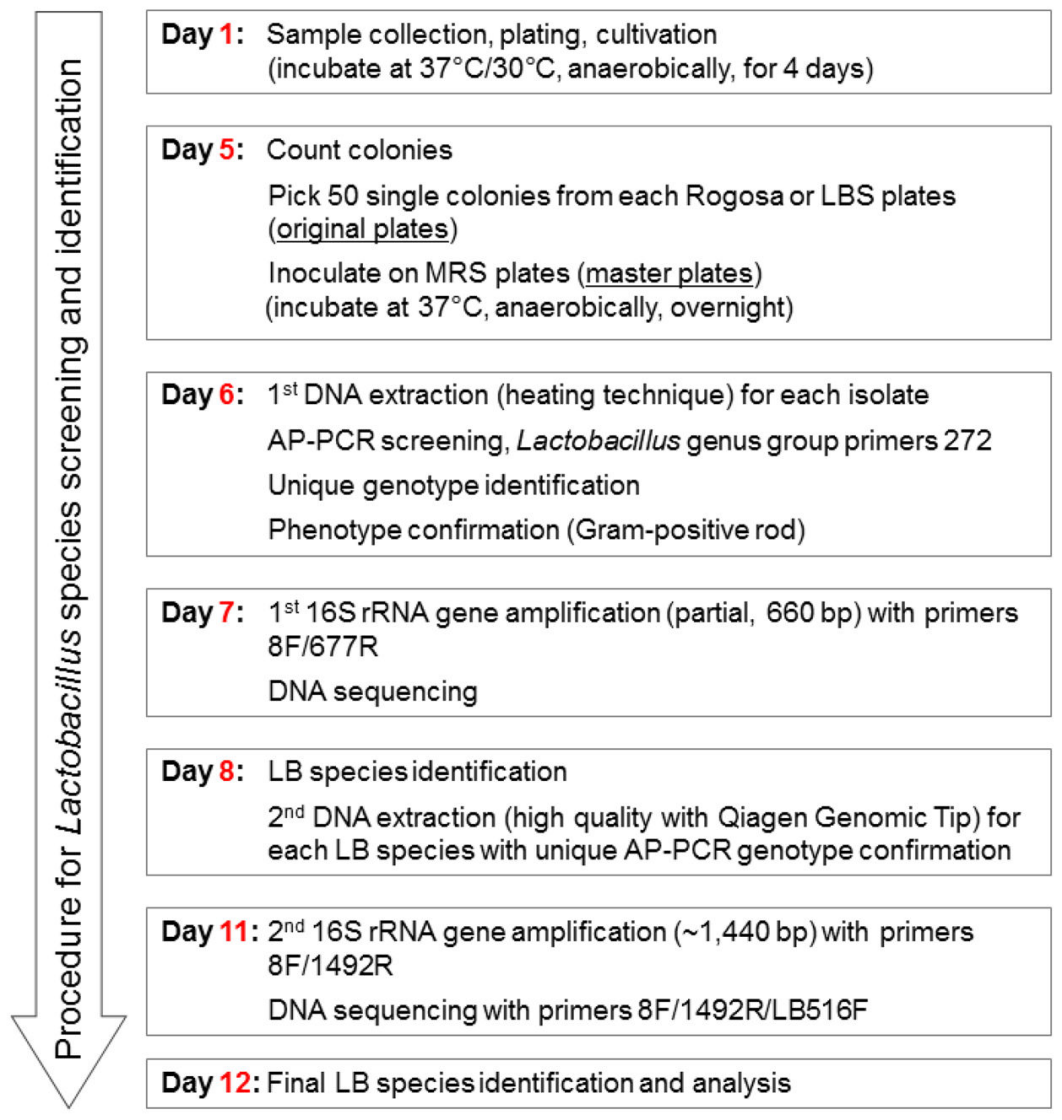
Flowchart of the culture-based component for *Lactobacillus* isolation, genotyping and species identification from clinical samples.

Previously, we observed that the saliva of caries-active mothers could harbor between 2 to 8 distinct genotypes of lactobacilli [29]. To estimate how many *Lactobacillus* genotypes are present in S-ECC children, we conducted another pilot study and found that, on average, children with S-ECC harbored 4 distinct genotypes, ranging from 1 to 6 [26]. In this study, we will first examine *Lactobacillus* colonization in the S-ECC children and compare to that of CF children; and access *Lactobacillus* diversity based on the bacterial samples grown on lactobacilli selective media (LBS). The primary *Lactobacillus* screening technique used will be arbitrarily primed polymerase chain reaction (AP-PCR) to genotype individual *Lactobacillus* isolates, followed by 16S rRNA gene sequencing for taxonomy assignment. The rapid screening protocol we proposed will take less than two weeks from *Lacto-bacillus* cultivation to identification (Figure 2).

#### 2.8.1. *Lactobacillus* Cultivation

Upon arrival of the bacterial sample, sterile glass beads (3 mm diameter) (Pyrex, Corning Incorporated, Corning, NY) will be added to the samples and mixed by vortexing for 30 s. Three 10-fold serial dilutions (10^−1^ to 10^−3^) of the sample will be used in order to obtain accurate colony forming unit (CFU) reads. The diluted samples (50 μl) will be plated on LBS medium, (Dickinson and Company, Sparks, MD) for recovery of lactobacilli using an Autoplate^®^ Spiral Plating System (Advanced Instruments, Inc., Norwood, MA). The samples will be incubated anaerobically (10% CO_2_, 10% H_2,_ and 80% N_2_) at 37°C (standard conditions) for 4 days, as well as in microaerophilic conditions (6% – 16% O_2_ and 2% – 10% CO_2_) at 30°C (alternative conditions) for 4 days to capture as many *Lactobacillus* species as possible [30]. For each culture plate, all CFU values will be counted and transformed logarithmically for normalization of the variation in the distribution and for statistical analysis.

#### 2.8.2. *Lactobacillus* Screening by AP-PCR Assay

After cultivation, 50 individual colonies per sample will be randomly selected with sterile toothpicks from the LBS plates and pure streaked on MRS agar plates (MRS, Becton Dickinson and Company, Sparks, MD). The MRS plates will be cultivated overnight anaerobically at 37 C. DNA will be extracted from the freshly grown culture by using a modified heating technique. Briefly, each of the bacterial samples will be transferred from the MRS agar plates, suspended in a 20 μl lysis solution (10 mM Tris-HCl buffer, 1 mM EDTA, 1% Triton X-100, pH 8.0) in a 96-well PCR plate, and incubated in a thermal cycler at 95°C for 10 min. The lysed samples will be immediately placed on ice for 3 – 5 min, and 1 μl of the supernatant will be used for PCR.

AP-PCR fingerprints will be obtained from each individual isolate. All PCR procedures will be performed using a Gene Amp PCR 9700 thermocycler (PE Applied Biosystems, Foster City, CA). The total volume of 50 μl consists of PCR buffer (20 mM Tris-HCl, 50 mM KCl, pH 8.4), 200 μM of each dNTP (Invitrogen, Carlsbad, CA), 7 mM MgCl_2_, 2.5 U of Taq DNA polymerase (Invitrogen), 100 pmol of primer of 272 (5′-AGCGGGCCAA-3′) [31], and 50 ng of DNA template. After an initial denaturation at 94°C for 3 min, the PCR reaction runs 45 cycles of 94°C for 30 s, 36°C for 30 s and 72°C for 1 min, followed by an extension of 5 min at 72 C. The PCR amplicons will be separated by electrophoresis in a 1.5% agarose gel run at 80 V for 3 h in Tris-borate-EDTA buffer. After electrophoresis, the gels will be stained for 15 min in ethidium bromide (10 mg/ml), followed by 15 min of destaining in water. The fingerprint images will be captured with a digital imaging system (Alpha IS-1000, Alpha Innotech Corp., San Leandro, CA). Unique AP-PCR fingerprint patterns will be identified and recorded. The isolates with unique AP-PCR fingerprinting patterns will be subjected to Gram staining for confirmation as Gram-positive regularly-shaped rods.

#### 2.8.3. 16S rRNA Gene Sequence for *Lactobacillus* Identification

Isolates displaying unique AP-PCR fingerprints and *Lactobacillus* phenotype by Gram staining are subjected to 16S rRNA gene sequencing analysis for species identification. The DNA templates isolated as described above will also be used for sequence analysis. A partial 16S rRNA gene will be amplified by PCR with universal bacterial primers 8F (5′-AGA GTT TGA TCC TGG CTC AG-3′) [32] and 677R (5′-CAC CGC TAC ACA TGG AG-3′) [33]. The 660-bp amplicon is designed for analysis of the hypervariable V1–V3 region. PCR amplification will be performed using the same GeneAmp PCR thermocycler in a total volume of 25 μl containing PCR buffer (20 mM Tris-HCl and 50 mM KCl, pH 8.4), 200 μM of each dNTP (Invitrogen, Carlsbad, CA), 2.5 mM MgCl_2_, 1.25 U of Taq DNA polymerase (Invitrogen), 0.5 pmol of each primer, and 25 ng of DNA template. After an initial denaturation at 94°C for 5 min, the PCR reaction will run 30 cycles at 94°C for 30 s, 55°C for 30 s and 72°C for 1 min, followed by an extension of 5 min at 72 C. The PCR products will be examined in 1% aga-rose gel, purified with the ExoSAP-IT PCR purification kit (Affymetrix, Santa Clara, CA), and sequenced with primer 8F (Genewiz, South Plainfield, NJ). Sequences will be compared to the 16S rRNA genes sequences from bacterial isolates available in the Ribosomal Database Project II (RDPII, http://rdp.cme.msu.edu/) [34] and in the Human Oral Microbiome Database (HOMD, http://www.homd.org) [35].

Taxonomic identification based on the 660 bp amplicon will be used to confirm membership in the *Lactoba-cillus* genus, and will, in most cases, provide a species-level assignment. Taxonomic identification to known species will be performed by RDF Sequence Match with an S_ab score greater than or equal to 0.95, or on the best blastn match to HOMD sequences with sequence identity greater than or equal to 98.5%. After initial screening, high-quality DNA of each representative *Lactobacillus* isolate will be obtained using Qiagen Genom-ic-tips 20/G (Qiagen Inc., Valencia, CA, USA). *Lactobacillus* species identification will be confirmed by sequencing the full-length 16S rRNA gene. A fragment of approximately 1.4 Kb will be amplified with universal primers 8F and 1492R [36], as described above, and sequenced with the same primers, plus a newly designed *Lactobacillus*-specific primer LB516F (5′-CGGCTAACTACGT-GCCAGCAG-3′) at Genewiz (South Plainfield, NJ). The three DNA sequence reads will be assembled with the BioNumerics suite (Applied Maths) and manually curated, and taxonomic identification to species level will be conducted.

### 2.9. Culture-Independent Assessment of *Lactobacillus* Diversity

In addition to culture-based analysis, the study plans to conduct a second analysis using a culture-independent approach to measure the overall abundance, diversity, and species affiliation of lactobacilli in the plaque samples. This method estimates both cultivable and uncultivable populations of lactobacilli, thus serving as a benchmark estimation of true diversity comparing the findings of the culture-based approach. To examine cultivation independent approach, multiple plaque samples will be collected from various sites: 1) pooled supragingival plaque samples; 2) pooled plaque samples within caries lesions, one from each of the four quadrants; 3) pooled plaque samples from non-caries fissures, one from each of the four quadrants. Genomic DNA will be obtained directly from each sample by methods that are well established in our lab [12] [37]. The DNA of pooled plaque sample will serve as a template for PCR amplification of almost the entire 16S rRNA locus (~1400 bp) with *Lactoba-cillus*-specific primers, Lacto F and Lacto R [26] [29] [38], to yield an approximate 232-bp fragment spanning the V2 – V3 region.

The 232-bp amplicon will be cloned into pCR4-TOPO vector and transformed into competent *E. coli* cells (Invitrogen). Fifty clones for each sample will be randomly selected and sequenced using vector primer T7 at the Genewiz sequencing facility (South Plainfield, NJ). Species assignment will be conducted as described above. The *Lactobacillus* phylotypes present in each sample will be tabulated, creating a taxonomic fingerprint for each sample. The final parameters for *Lactobacillus* diversity analysis will include the numbers and distribution of *Lactobacillus* species/phylotypes (distinct 16S rRNA gene sequences), the abundance and richness of *Lactoba-cillus* species, as well as phylotypes for different groups of samples.

### 2.10. Data Management and Statistical Analysis

The primary outcome of the culture-based method will be the level of *Lactobacillus* colonization measured by colony forming units (CFU) for each sample. The quantitative results will be analyzed against other independent variables including caries score, children’s medical history, oral hygiene behavior, and family socio-demographic factors.

The second outcome of the study will be the number of unique *Lactobacillus* genotypes per individual as determined by AP-PCR. All of the AP-PCR fingerprint profiles will be examined; pair-wise similarity (inter- and intra-lactobacilli isolates) will be calculated based on the Dice coefficient. Hierarchical cluster analyses will be performed using BioNumerics 6.0 program (Applied Maths, Austin, TX). The results will be compared between S-ECC and CF children, and among different types of clinical samples, such as total plaque versus carious lesion and sound fissure.

The third outcome of the study will be the abundance and distribution of *Lactobacillus* species between S-ECC and CF groups. For *Lactobacillus* sequence analysis, the Chi-square test will be used to compare phylo-genetic differences between the two groups. Species diversity and richness will be evaluated using Chao1, abundance-based coverage estimator (ACE), rarefaction curves, rank abundance and diversity indices, Shannon (*H*′), as well as Good’s percent coverage [39].

The fourth outcome of the study will involve carrying out the delineation of caries-associated genetic elements *Lactobacillus* strains obtained from S-ECC and CF children. Intra- and inter-species comparisons with advanced classification algorithms will be performed to identify the loci specific to all S-ECC *Lactobacillus* species and to all CF *Lactobacillus* species. The analysis will focus on oral *Lactobacillus* genomes and genetic loci associated with niche specialization. Further genomic comparisons with environmental and food-associated lactobacilli will allow us to deduce species-specific genetic signatures for the oral niche.

Finally, all of the data, socio-demographics, medical history, questionnaires, dental examine, *Lactobacillus* isolates, specie identifications, as well as documents and images will be entered, stored, and managed by a custom-designed program for this project using FileMaker Pro 12 program (FileMaker, Inc. Santa Clara, CA) for this study. In addition, lab materials will also be labeled with 2D barcodes to protect subject identification, prevent data entry errors, and track sample process. Information in the dataset can be exported into Microsoft Excel files for statistical analysis.

SPSS Statistics software v22.0 (IBM Corp., Somers, NY) and STATA v12.1 (Stata Corp, College Station, TX) will be used for the data analyses. ANOVA and nonparametric independent tests will be used for continuous variables (CFU counts, age, and caries score). Chi-square and Pearson Correlation tests will be used for comparisons of clinical data (gender, mode of delivery, use of antibiotics, and oral hygiene behavior) with *Lactobacillus* colonization between S-ECC and CF groups and between the two different study methods as for culture-based versus molecular-based approach.

## 3. Discussion

S-ECC is a serious public health concern as a result of its early onset, rapidly clinical progression, high treatment cost and negative impact on oral health-related quality of life of young children. The pathology of S-ECC is highly complex. The primary etiologic agents of dental caries associated with the disease are the mutans streptococci, particularly, *Streptococcus mutans* [40]. *Lactobacillus* species and other non-mutans acidogenic and acid-tolerant bacteria [41] are also known as significant etiologic agents [5] [26]. Epidemiological studies have shown a wide range of *Lactobacillus* colonization in newborn, preschool children, adolescent, and adult populations, but not all children or adults who are colonized by lactobacilli are susceptible to dental caries. Lac-tobacilli-dentinal caries correlation has been discovered for decades, even earlier than the discovery of the *S. mutans*-caries linkage [42] [43]. Valid and reliable measures for assessment of lactobacilli colonization and prediction of caries outcome are still lacking. Clinically, new treatment interventions of S-ECC require the regular evaluation of cariogenic bacterial colonization in the oral cavity. Although oftentimes a few chair-side commercial test kits are available in dental clinics, results of discrepancy from those test kits cannot adequately predict children at high risk for caries. Future S-ECC intervention and treatment would be more effective if we can adequately identify *Lactobacillus* species and define their genetic specification and pathophysiologic characteristics associated with S-ECC. Importantly, the existence of a link between specific *Lactobacillus* species and S-ECC remains to be elucidated.

Based on selective culture media, previous studies of caries-associated lactobacilli have been mostly quantitative. The culture-based approaches can be time- and labor-intensive, and results can vary widely based on colony morphology or counts on the particular medium or cultivation method used. On the other hand, using culture-independent methods for taxonomic differentiation of lactobacilli can be challenging as well because of a vast group of Gram-positive bacilli sharing similar morphological profiles as well as the molar percentage of guanine-cytosine content (% G + C). Furthermore, there are very few definitive *Lactobacillus* species-specific 16S rRNA gene primers/probes, thus creating more obstacles against the use of advanced molecular-based techniques to determine lactobacilli diversity, abundance and taxonomical affiliation.

The study proposes a well-designed methodology aimed to assure that the sampling depth will be sufficient to find most *Lactobacillus* species associated with dental caries. Unlike most culture-based studies of the microbi-ota of S-ECC, this protocol utilizes a dual approach that utilizes both culture-based and culture-independent approaches. The culture-based component relies on a *Lactobacillus* selective medium that eliminates the background growth of other acid-tolerant oral bacteria, and can be completed, from sample plating to lactobacilli screening and isolation, in 10 to 12 working days for each subject. The workflow can be staggered for multiple subjects, allowing a trained team of dentists and lab technicians to process 5 – 10 subjects in one day. *Lactoba-cillus* strains isolated based on the selective medium can also serve as source materials for the whole genome sequencing and comparative genomics leading to the identification of niche-specific loci. The culture-independent component can be completed in 5 to 7 days from sample collection, genomic DNA purification to retrieval of the *Lactobacillus* 16S rRNA sequences for each subject. Up to 8 subjects can easily be processed simultaneously. Moreover, the culture-independent component relies on a new set of *Lactobacillus*-specific primers which allows for the detection of lactobacilli in plaque samples, even though they are present in low numbers. That all elements of the study will incorporate a custom-designed barcoding identification and tracking system and a user friendly interface will greatly enhance the efficiency and accuracy of samples process and data management.

## 4. Conclusion

In summary, the novel culture-dependent and culture-independent approach will enable us to determine the complexity and the role of lactobacilli played in S-ECC. From a clinical perspective, *Lactobacillus* function- and niche-specific cariogenic variables can serve as antimicrobial targets for new therapeutic treatment for caries and help facilitate the development of new tools for future caries risk assessments.

## Abbreviations

S-ECC: severe early childhood caries
CF: caries free
dmft/dmfs: decay, filled or missing (due to caries) tooth/tooth surfaces in primary dentition
MS: mutans streptococci
LB: lactobacilli
CFU: colony forming units
LBS: lactobacilli selection medium
LDT: liquid dental transport medium
AP-PCR: arbitrarily primed polymerase chain reaction
RDP: Ribosomal Database Project
HOMD: Human Oral Microbiome Database
